# Inosine Exerts Glioprotective Effects against Lipopolysaccharide-Induced Damage in Primary Astrocyte Cultures: Role of the Adenosinergic System

**DOI:** 10.1021/acsomega.6c02414

**Published:** 2026-09-02

**Authors:** Nathalia S. Pedra, Mayara S. S. de Aguiar, Fernanda C. Teixeira, Julia E. de Mello, Natália P. Bona, Larissa M. da Silveira, Thais M. da Rosa, Juliane T. Saraiva, William S. Simões, Daniela B. R. Leal, Rejane G. Tavares, Francieli M. Stefanello, Roselia M. Spanevello

**Affiliations:** † Center for Chemical, Pharmaceutical and Food Sciences, Graduate Program in Biochemistry and Bioprospecting − Laboratory of Neurochemistry, Inflammation and Cancer, Federal University of Pelotas, Pelotas, RS 96160-000, Brazil; ‡ Center for Chemical, Pharmaceutical and Food Sciences, Graduate Program in Biochemistry and Bioprospecting – Biomarkers Laboratory, Federal University of Pelotas, Pelotas, RS 96160-000, Brazil; § Department of Microbiology and Parasitology – Experimental and Applied Immunobiology Laboratory, Federal University of Santa Maria, Santa Maria, RS 97105-900, Brazil

## Abstract

Inosine is a purine nucleoside derived from adenosine metabolism that has been associated with neuroprotective and anti-inflammatory properties. The present study investigated the effects of inosine against lipopolysaccharide (LPS)-induced damage in primary astrocyte cultures and explored the potential contribution of adenosinergic signaling to these responses. LPS exposure impaired astrocyte viability and proliferation, altered cholinergic and purinergic enzyme activities, disrupted redox homeostasis, and increased the level of IL-6 release. Inosine treatment attenuated these alterations, restoring cell viability and proliferation, modulating acetylcholinesterase and ectonucleotidase activities, reducing oxidative damage, and preventing the LPS-induced increase in IL-6 levels. Mechanistic studies using pharmacological modulation of the adenosinergic system revealed that blockade of adenosine receptors interfered with some of the antioxidant effects of inosine, particularly those related to antioxidant enzyme activity, whereas its effects on cytokine modulation remained unchanged. Additional experiments using selective A2A receptor antagonists provided preliminary evidence that A2A receptor signaling may be involved in the antioxidant-related effects observed under inosine-treated conditions. Collectively, these findings demonstrate that inosine exerts multitarget protective effects in astrocytes, modulating oxidative stress, neurotransmission-related pathways, and inflammatory responses. These results support its potential as a promising strategy for restoring astrocyte homeostasis under neuroinflammatory conditions.

## Graphical abstract

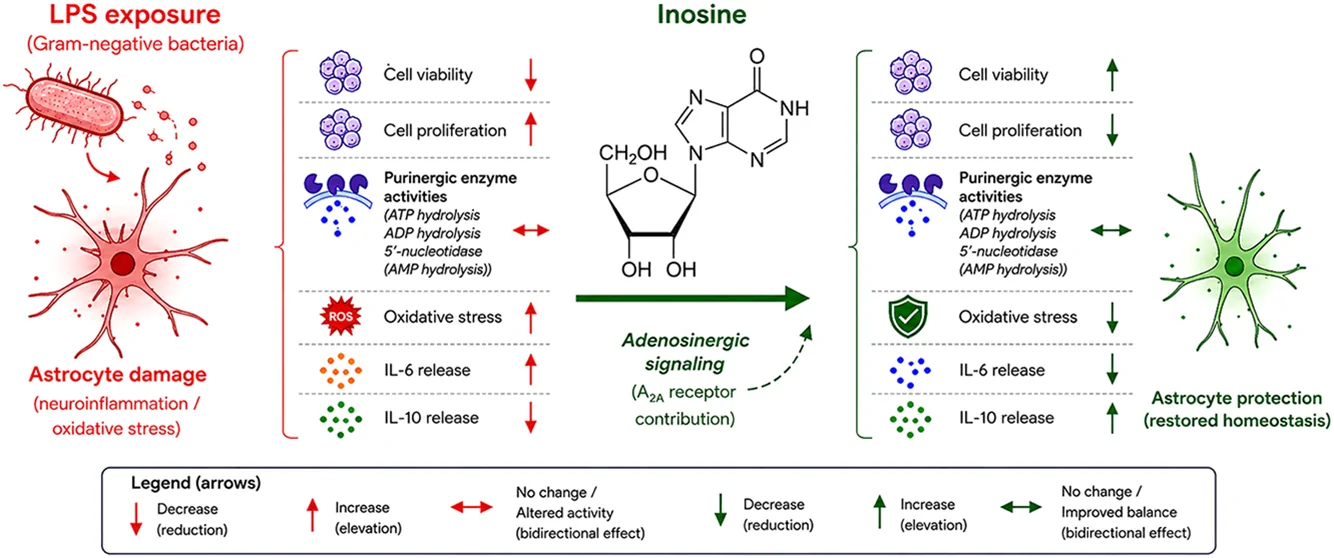

## 1 Introduction

Inosine is a purine nucleoside formed mainly from adenosine by the enzyme adenosine deaminase and acts as a molecular messenger in cell signaling pathways.^1^ There is a growing body of evidence supporting significant biological effects of inosine, including anti-inflammatory and neuroprotective actions.^2^ Due to these properties, inosine has been proposed as a promising candidate for exerting a therapeutic impact on mechanisms implicated in the development and progression of neurological disorders.^1–5^ These promising effects appear to involve the interaction between inosine and adenosine receptors, which generally show lower binding affinity compared to adenosine. While direct binding to the A2B receptor has not been confirmed, strong evidence indicates that inosine functionally engages all four adenosine receptor subtypes.^5^

Neuroinflammation represents a complex immune response within the central nervous system, playing a crucial role in the development of brain diseases.^6^ Among the key contributors to this intricate process are the astrocytes. These remarkable cells release various signaling molecules, including cytokines, reactive oxygen species (ROS) and reactive nitrogen species (RNS), and adenosine triphosphate (ATP), which can either exacerbate or mitigate neuroinflammation.^7–9^ In fact, the interconnection between oxidative stress and neuroinflammation is well-established. In brain injury, the excessive production of ROS/RNS, coupled with a decline in antioxidant defenses, can lead to chronic astrogliosis.^10^ This condition involves a heightened reactivity of astrocytes, contributing to a sustained generation of free radicals and prolonged release of proinflammatory cytokines, culminating in neuronal damage and death.^11^

In addition, purinergic signaling is mediated by extracellular adenine nucleotides and nucleosides such as ATP, ADP, and adenosine.^12^ In the brain, purinergic receptors are involved in the regulation of the inflammatory response in a complex manner, including the activation of the inflammasome leading to the synthesis and liberation of proinflammatory cytokines, activation and migration of microglia, and alteration in astrocyte function.^13^ In response to physiological and pathological stimuli, astrocytes can release ATP into the extracellular milieu, which can be rapidly hydrolyzed into adenosine by ectonucleotidases such as NTPDase and 5́-nucleotidase, or activate purine receptors.^14^ Considering that the increase in extracellular ATP acts as a proinflammatory mediator through the P2X7 receptor, the modulation of ectonucleotidase activities in astrocytes can play a crucial central role in controlling exacerbated neuroinflammation.^15^

The dynamic interplay among astrocytes, oxidative stress, neuroinflammation, and neuronal damage underscores the complexity of these processes and the importance of further research into potential therapeutic interventions. Understanding the precise mechanisms by which astrocytes contribute to neuroinflammation is of paramount importance, as it offers potential possibilities for the development of innovative therapeutic strategies addressing neurological disorders where neuroinflammation is a key factor. The induction of inflammation using lipopolysaccharide (LPS) has been extensively employed to replicate neuroinflammatory conditions and serves as a screening tool for new neuroprotective agents.^16,17^ Therefore, the aim of this study was to investigate the role of inosine in modulating purinergic and cholinergic signaling, as well as oxidative stress in astrocytes exposed to LPS, and the involvement of the adenosinergic system.

## 2 Results and Discussion

### 2.1 Inosine Reverses LPS-Induced Alterations of Astrocyte Viability and Proliferation

Initially, we evaluated the cytotoxic profile of inosine in primary cultures of astrocytes at concentrations of 12.5, 25, 50, and 100 μM for 48 and 72 h. No alterations in cell viability or proliferation were observed when compared to untreated cells, revealing that inosine is not harmful to healthy brain cells at these concentrations (Figure 1). This finding is consistent with previous studies reporting that inosine (1–10 mM) does not affect oligodendrocyte viability *in vitro*.^18^

**Figure 1:**
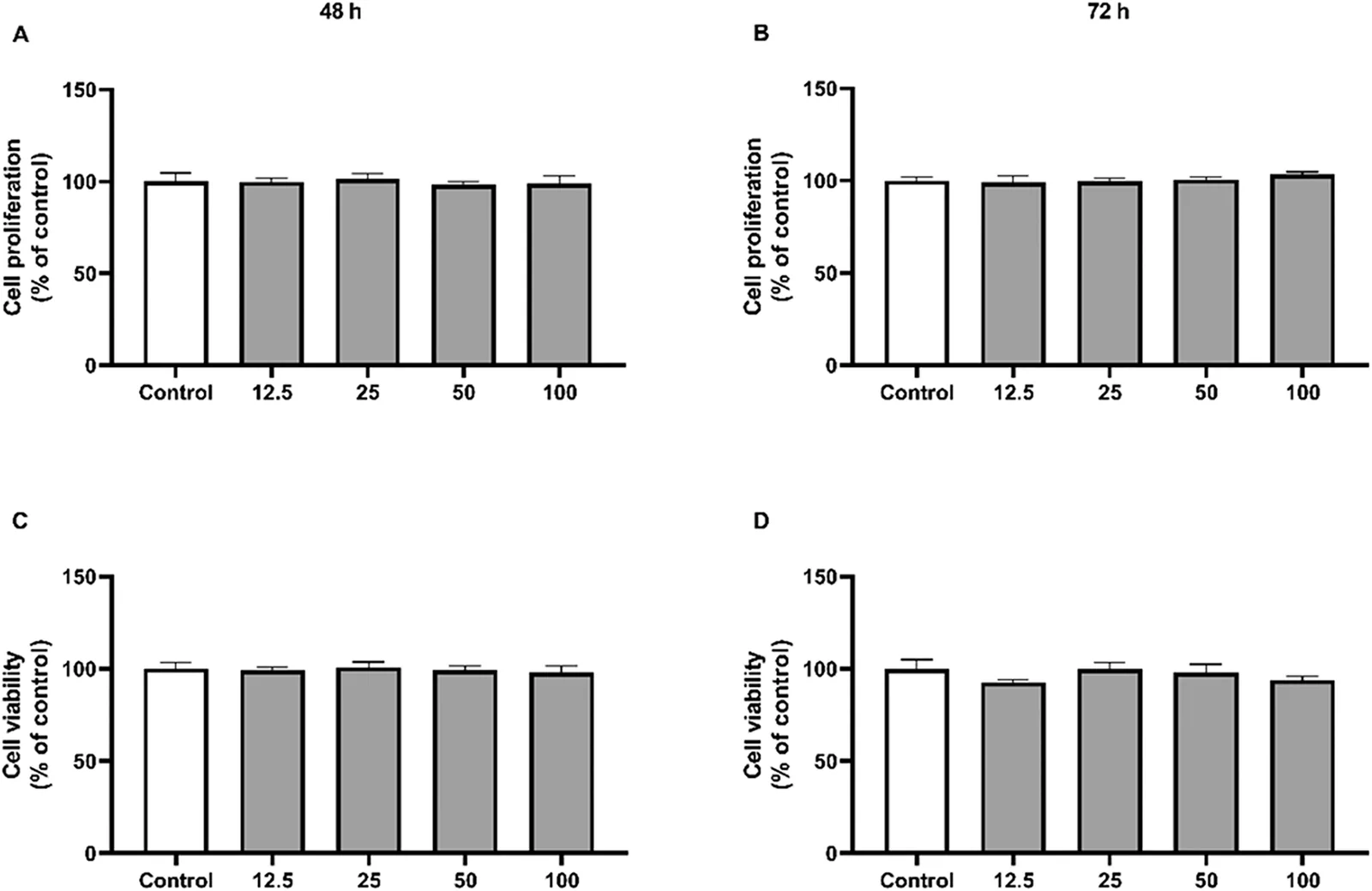
Cytotoxic evaluation of inosine in primary culture of astrocytes. Cultures were exposed to inosine (12.5–100 μM). Cell proliferation was assessed using the SRB assay after 48 h (A) and 72 h (B), whereas cell viability was evaluated using the MTT test after 48 h (C) and 72 h (D) of treatment. The experiments were performed in triplicate. Data were analyzed by one-way ANOVA followed by Tukey’s post hoc test, and are presented as mean ± SEM.

In the next set of experiments, we investigated the potential of inosine to reverse LPS-induced cytotoxicity in an astrocyte culture. As demonstrated in Figure 2A and B, LPS induced an increase in cell proliferation at both evaluated times (about 65% and 55% after 48 and 72 h, respectively). In contrast, treatment with inosine for 48 and 72 h reduced cell proliferation , suggesting a glioprotective effect. Furthermore, exposure to LPS reduced astrocyte viability by approximately 30% after 48 h when compared with control cells. However, treatment with inosine prevented these deleterious effects, increasing cell viability by about 30% when compared to astrocytes challenged with LPS (Figure 2C and D). In addition, representative micrographs showed marked morphological alterations after LPS exposure (Figure 2E). Qualitative inspection of the images suggested that, compared with the control group, LPS-treated cultures contained more enlarged and highly refractive cells, showed a less homogeneous cellular distribution, and exhibited more apparent cellular clustering. These morphological features may be compatible with a reactive phenotype, although astrocyte activation was not directly confirmed by using specific markers. In inosine-treated cultures, these alterations appeared to be less pronounced, with a more homogeneous cellular organization and fewer cells displaying an apparently reactive morphology. These observations suggest a possible attenuation of LPS-associated morphological changes; however, they should be interpreted cautiously, because they were based on qualitative image assessment. At 72 h, no clear qualitative differences were observed among the experimental groups.

**Figure 2:**
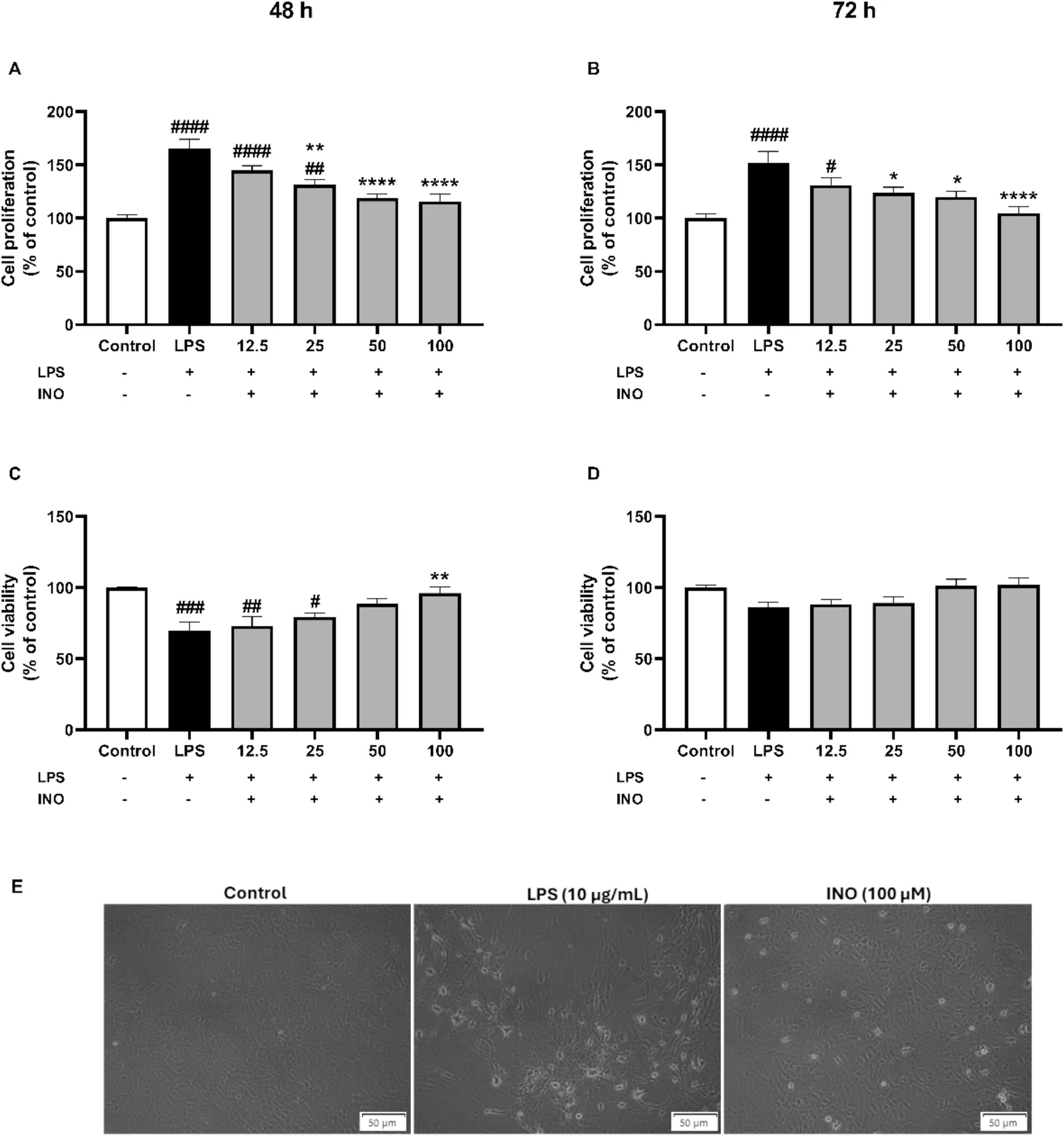
Effect of inosine (INO) on astrocyte proliferation (A, B) and viability (C, D) following LPS exposure. After 3 h of exposure to LPS (10 μg/mL), the cells were treated with INO (12.5–100 μM) for 48 h (A, C) and 72 h (B, D). Representative optical microscopy images (E) show astrocyte morphology in the Control, LPS, and LPS + INO groups. The experiments were performed in triplicate. Data were analyzed by one-way ANOVA followed by Tukey’s post hoc test and are presented as mean ± SEM. ^#, ##, ###, ####^ Significant difference compared to control cells (*P* < 0.05, *P* < 0.01, *P* < 0.001, and *P* < 0.0001, respectively). *, **, **** Significant difference compared to the LPS group (*P* < 0.05, *P* < 0.01, and *P* < 0.0001, respectively). Photograph courtesy of Nathalia S. Pedra. Copyright 2025.

In line with previous findings,^19,20^ it was demonstrated that exposure to LPS induces proliferation and reduces the viability of astrocytes. LPS activates a cascade of intracellular signaling events through the Toll-like receptor 4 (TLR4) located on the astrocyte membrane. Among the key pathways activated is the NF-κB signaling mediated via the MyD88-IRAK-TRAF6-TAK1 complex. The transcription factor NF-κB plays a central role in regulating the expression of proinflammatory genes by translocating to the nucleus and initiating a range of inflammatory responses.^16^ Additionally, it has been proposed that the LPS-induced increase in cell proliferation is regulated through activation of the PI3K/Akt pathway.^21^

Inosine treatment protected against LPS-induced alterations in cell viability and proliferation. These protective effects can be attributed to the multiple biological activities of inosine. As demonstrated in the present study, these include anti-inflammatory effects through modulation of ectonucleotidase and acetylcholinesterase (AChE) activities, and antioxidant properties, as evidenced by the reduction of reactive species and the regulation of antioxidant enzyme activity in response to LPS-induced damage.

### 2.2 Evaluation of Acetylcholinesterase (AChE) Activity in Astrocytes Exposed to LPS and Inosine

The impact of inosine on the modulation of AChE activity was investigated in cells exposed to LPS. As observed in Figure 3, LPS induced an increase in AChE activity after 48 h (about 60%) and 72 h (about 80%) in astrocytes when compared to control cells. However, treatment with 100 μM inosine decreased AChE activity in astrocytes by approximately 35% after 48 h (Figure 3A). Inosine did not significantly prevent the deleterious effects induced by LPS after 72 h (Figure 3B).

**Figure 3:**
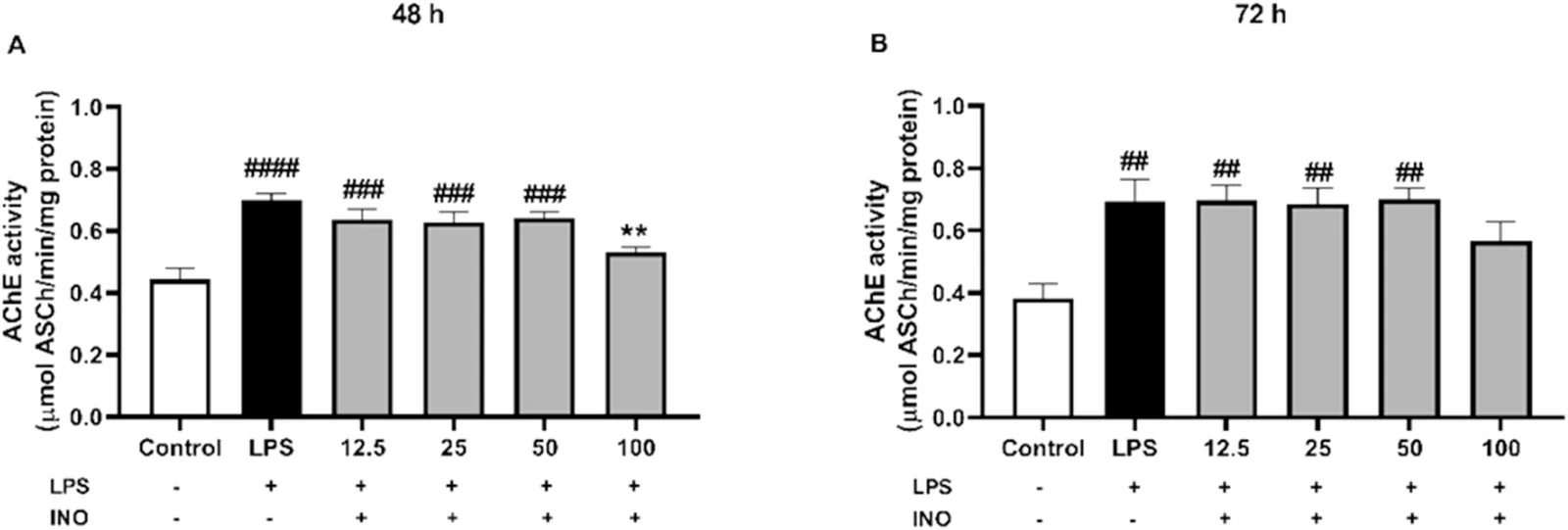
Acetylcholinesterase (AChE) activity in primary astrocyte cultures exposed to LPS (10 μg/mL) and inosine (INO; 12.5–100 μM) for 48 h (A) and 72 h (B). The experiments were performed in triplicate. Data were analyzed by one-way ANOVA followed by Tukey’s post hoc test and are presented as mean ± SEM. ^##, ###, ####^ Significant difference compared to control cells (*P* < 0.01, *P* < 0.001, and *P* < 0.0001, respectively). ** Significant difference compared to the LPS group (*P* < 0.01).

AChE plays a crucial role in rapidly breaking down acetylcholine into acetic acid and choline. Apart from its catalytic function, AChE is also involved in various other processes such as cell adhesion, proliferation, neurite outgrowth, and glial activation.^22^ In the brain, acetylcholine can act as both an astrocytic mitogen and an anti-inflammatory mediator.^23^ Indeed, mice treated with a nicotinic acetylcholine receptor agonist showed reduced nuclear translocation of NF-κB, resulting in decreased expression and secretion of TNF-α.^24^

Acetylcholine has been shown to protect against LPS-induced neuronal injury by modulating the release of proinflammatory mediators, including TNF-α and IL-1β, from microglia.^25^ In the present study, LPS increased AChE activity in astrocytes, likely reducing acetylcholine levels and amplifying the inflammatory response. The modulatory effect of inosine on AChE activity has also been reported in the cerebral cortex and hippocampus in Alzheimer’s disease models.^26,27^ Additionally, inosine has been shown to increase the expression of choline acetyltransferase (ChAT), the enzyme responsible for acetylcholine biosynthesis, in both the cerebral cortex and hippocampus of rats.^26^ Together, these findings suggest that modulation of cholinergic signaling may be associated with the neuroprotective profile of inosine in astrocytes. Although the present study did not directly investigate causal mechanisms linking AChE activity to the protective effects of inosine, the enzymatic changes observed here provide indirect mechanistic support for the involvement of cholinergic pathways in the modulation of astrocyte inflammatory responses.^19,20^

### 2.3 Inosine Modulates Changes in ATP, ADP, and AMP Hydrolysis Induced by LPS

LPS induced an increase in ATP hydrolysis in astrocytes after 48 and 72 h of exposure compared to control cells, reaching approximately 150% and 45%, respectively (Figure 4A and B). Although inosine did not significantly affect ATPase activity after 48 h of treatment, it markedly attenuated the LPS-induced increase after 72 h, reducing enzyme activity by up to 40%. Regarding ADP hydrolysis, LPS induced an increase at 48 h by approximately 120%, but a decrease at 72 h by about 55%, compared to control astrocytes (Figure 4C and D). A significant reduction in AMP hydrolysis was observed in LPS-challenged astrocytes (50% at 48 h and 60% at 72 h) when compared to control cells (Figure 4E, F). In contrast, treatment with inosine at a concentration of 100 μM reversed these changes, reaching levels of ADPase and AMPase activity similar to those shown by control cells.

**Figure 4:**
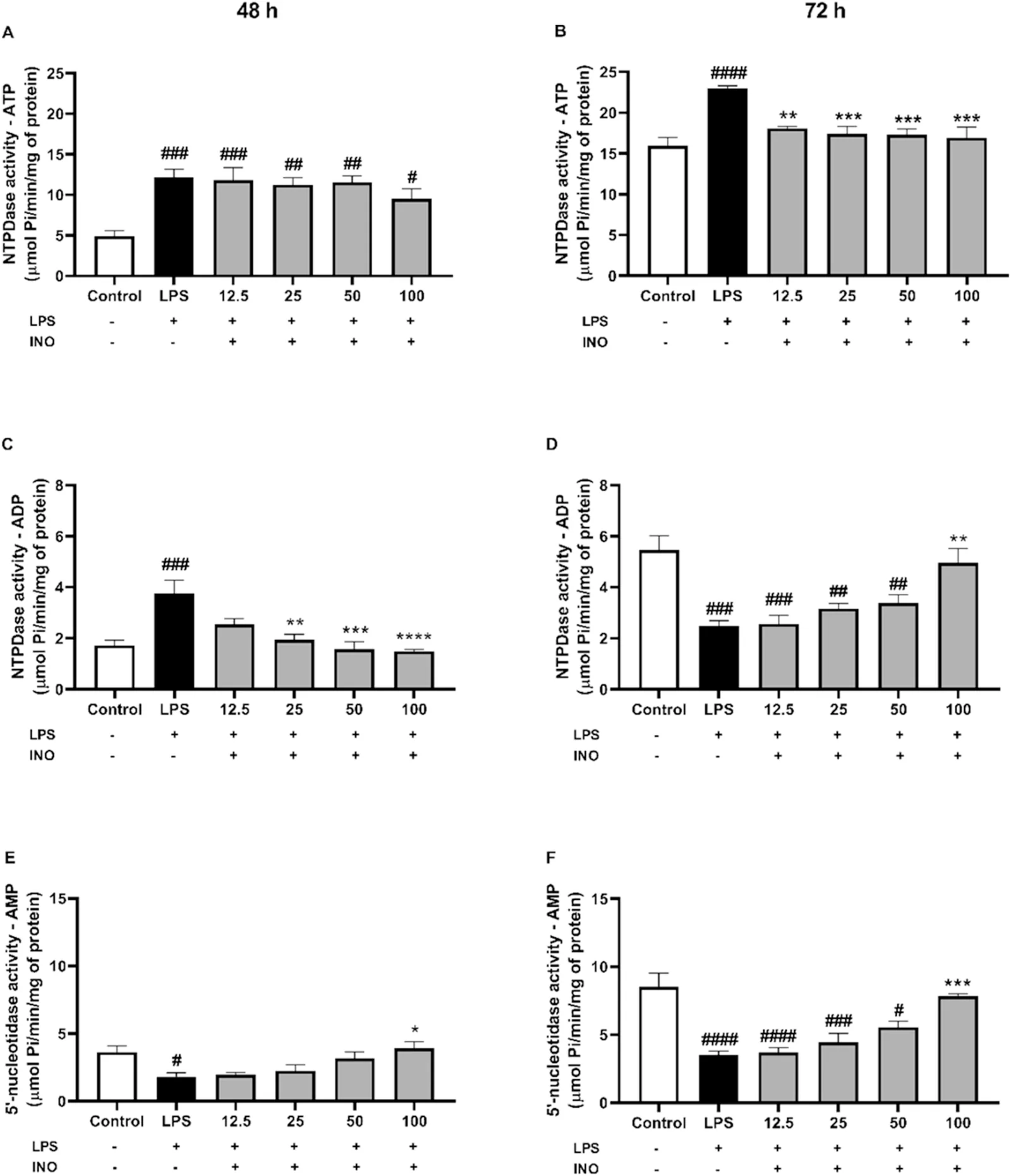
Nucleotide hydrolysis in astrocytes exposed to LPS (10 μg/mL) and inosine (INO; 12.5–100 μM) after 48 h (A, C, E) and 72 h (B, D, F). (A, B) NTPDase activity using ATP as substrate; (C, D) NTPDase activity using ADP as substrate; and (E, F) 5′-nucleotidase activity using AMP as substrate. The experiments were performed in triplicate. Data were analyzed by one-way ANOVA followed by Tukey’s post hoc test and are presented as mean ± SEM ^#, ##, ###, ####^ Significant difference compared to control cells (*P* < 0.05, *P* < 0.01, *P* < 0.001, and *P* < 0.0001, respectively). *, **, ***, **** Significant difference compared to the LPS group (*P* < 0.05, *P* < 0.01, *P* < 0.001, and *P* < 0.0001, respectively).

The increase in ATP hydrolysis following LPS exposure may reflect elevated extracellular ATP levels under neuroinflammatory conditions.^28^ As a damage-associated molecular pattern (DAMP), ATP activates glial cells through P2X7 receptor stimulation in astrocytes. This activation promotes membrane remodeling, release of IL-1β–enriched extracellular vesicles, and an increase in free radicals, further amplified by proinflammatory cytokines such as IL-1β and TNF-α. Together, these events contribute to the progression of neuroinflammation and neurodegeneration.^28,29^

The increase in ATP hydrolysis may lead to a decrease in ADP concentrations, resulting in a reduced demand for further ADP hydrolysis, which is consistent with the data observed at 72 h. In contrast, at 48 h, an increase in ADP hydrolysis was detected, suggesting a temporal progression in ADP degradation. Initially, enhanced ATP hydrolysis raises ADP levels, which subsequently triggers a greater demand for ADP hydrolysis. Subsequently, the increased activity of NTPDase results in a significant reduction in extracellular ADP levels, which in turn may reduce the enzyme’s activity due to the limited availability of its substrate. This process ultimately leads to an increased production of AMP. However, 5′-nucleotidase activity was reduced at both experimental times in the LPS group, which may impair adenosine production and potentially contribute to diminished neuroprotective and neuromodulatory signaling. Although these findings do not establish a direct causal relationship between ectonucleotidase modulation and inosine-mediated protection, they provide indirect mechanistic evidence supporting the involvement of purinergic homeostasis in the astrocytic response to inflammatory injury.^19,20,30,31^

Although the effects of adenosine can vary depending on the receptor subtype activated, recent evidence has shown that in human astrocytes, its inhibitory effect on cell proliferation occurs independently of receptor activation.^32^ Notably, inosine exhibited a protective effect against LPS-induced alterations in ectonucleotidase activity. Although the precise mechanisms underlying inosine’s biological effects are not fully understood, and no specific receptor for inosine has been identified, several studies have demonstrated its interaction with adenosine receptors, which are involved in both presynaptic and postsynaptic neuromodulatory processes.^1,3,33^

### 2.4 Inosine Reduces LPS-Induced Oxidative Damage and Improves Astrocyte Antioxidant Defense Systems

The results of the oxidative stress are described in Figures 5 and 6. An increase in the levels of ROS (160% at 48 h and 340% at 72 h; Figure 5A and B) and nitrites (130% at 48 h and 60% at 72 h; Figure 5C and D) was observed in astrocytes after exposure to LPS at both time points when compared to control cells. Inosine was able to reverse all changes induced by LPS, reducing ROS levels by up to 167% and 320%, and nitrites by up to 190% and 60% after 48 and 72 h of cultivation, respectively. Regarding thiol content (SH), exposure of astrocytes to LPS reduced SH levels by approximately 55% and 60% at 48 and 72 h of culture, while treatment with inosine was able to reverse these changes.

**Figure 5:**
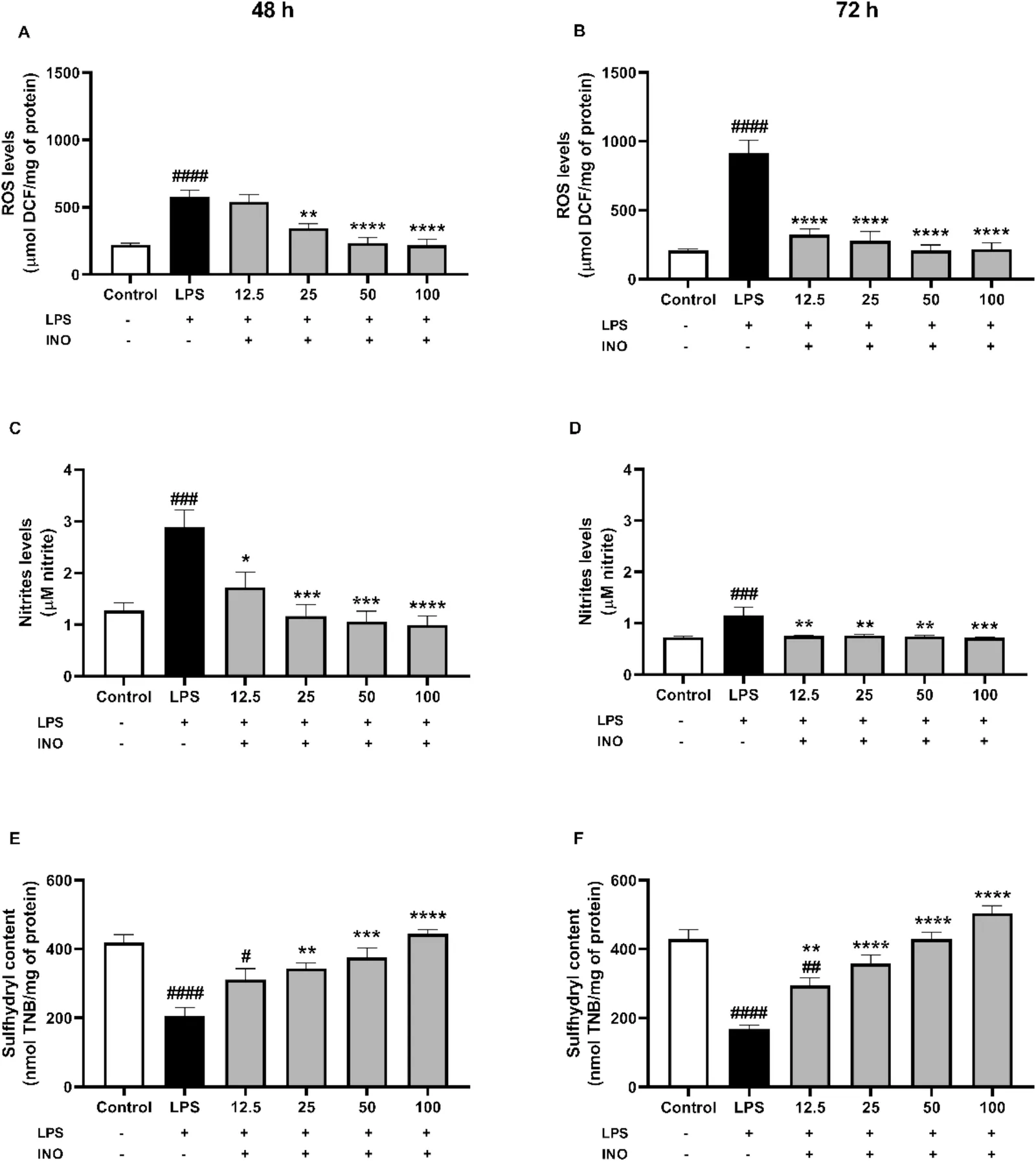
Reactive oxygen species (ROS) (A, B) and nitrite levels (C, D), and total sulfhydryl (SH) content (E, F) in primary astrocyte cultures exposed to LPS (10 μg/mL) and inosine (INO; 12.5–100 μM) after 48 h (A, C, E) and 72 h (B, D, F). The experiments were performed in triplicate. Data were analyzed by one-way ANOVA followed by Tukey’s post hoc test and are presented as mean ± SEM. ^#,##,###, ####^ Significant difference compared to control cells (*P* < 0.05, *P* < 0.01, *P* < 0.001, and *P* < 0.0001, respectively). *, **, ***, **** Significant difference compared to the LPS group (*P* < 0.05, *P* < 0.01, *P* < 0.001, and *P* < 0.0001, respectively).

**Figure 6:**
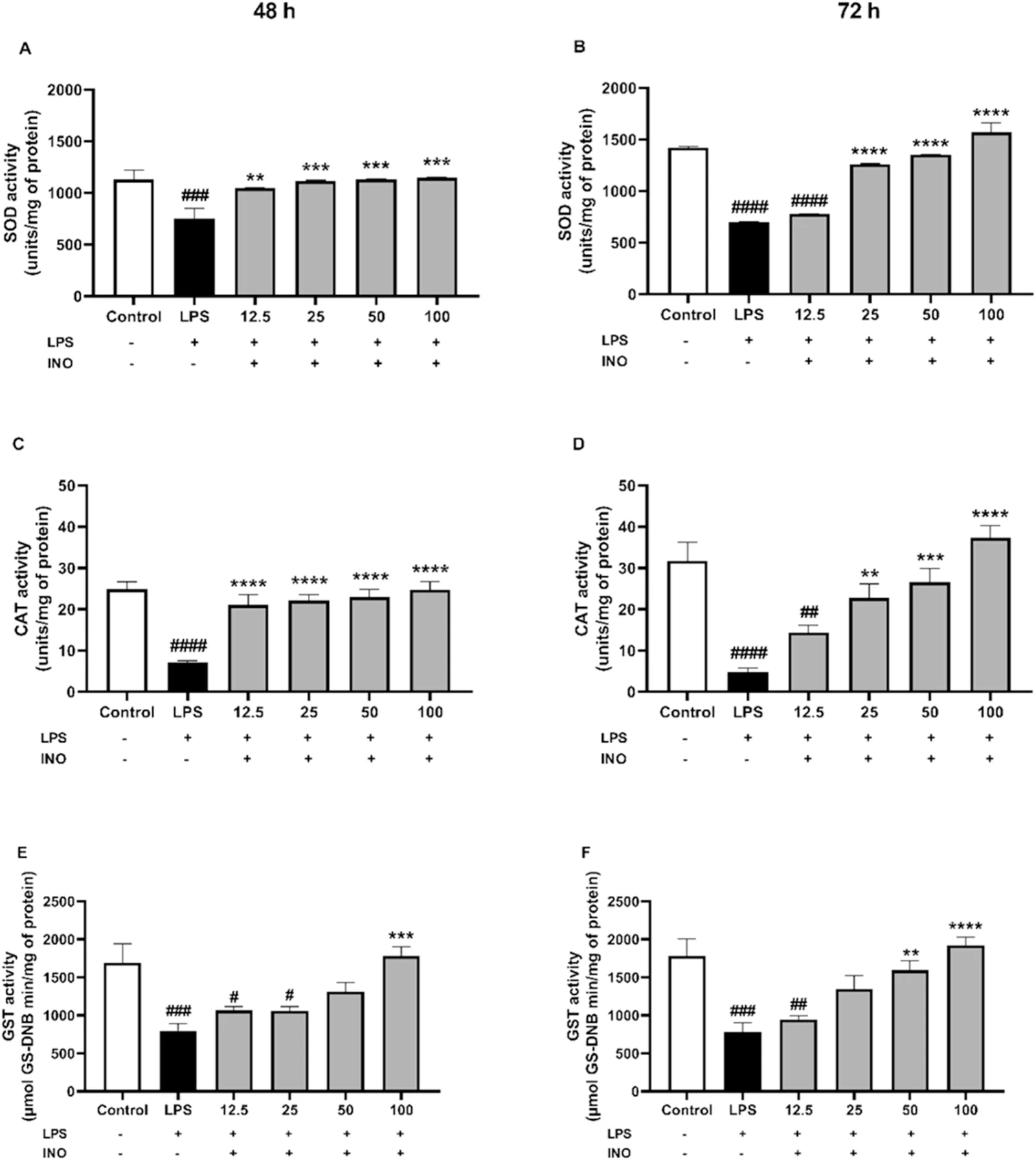
Superoxide dismutase (SOD) activity (A, B), catalase (CAT) activity (C, D), and glutathione S-transferase (GST) activity (E, F) in primary astrocyte cultures exposed to LPS (10 μg/mL) and inosine (INO; 12.5–100 μM) after 48 h (A, C, E) and 72 h (B, D, F). The experiments were performed in triplicate. Data were analyzed by one-way ANOVA followed by Tukey’s post hoc test and are presented as mean ± SEM. ^#,##,###, ####^ Significant difference compared to control cells (*P* < 0.05, *P* < 0.01, *P* < 0.001, and *P* < 0.0001, respectively). **, ***, **** Significant difference compared to the LPS group (*P* < 0.01, *P* < 0.001, and *P* < 0.0001, respectively).

LPS reduced the activity of antioxidant enzymes SOD, CAT, and GST by approximately 35%, 70%, and 55%, respectively, compared to healthy astrocytes after 48 h (Figure 6A, C, E). Similar results were found after 72 h of cultivation, where LPS caused a significant reduction in the activity of antioxidant enzymes of approximately 50% for SOD (Figure 6B), 85% for CAT (Figure 6D), and 55% for GST (Figure 6F). Treatment with inosine reversed the effects induced by LPS, increasing the activity of these enzymes by up to 55%, 88%, and 60%, respectively.

LPS-induced oxidative damage in astrocytes is evidenced by increased levels of oxidative markers, such as ROS and nitrites, alongside a reduction in both nonenzymatic (e.g., sulfhydryl groups, SH) and enzymatic (e.g., SOD, CAT, and GST) antioxidant defenses. LPS induces an increase in TNF-α levels, and upon binding of TNF-α to TNF receptor 1 (TNFR1), the formation of signaling complex I is initiated, a well-established step in the activation of downstream inflammatory pathways. This complex subsequently triggers the activation of the NF-κB pathway, facilitating its binding to DNA and promoting ROS generation. Such activity is achieved by upregulating the expression and activity of inducible nitric oxide synthase (iNOS) and NADPH oxidase 2 (NOX2) in glial cells like astrocytes and microglia. Additionally, nitrites serve as an indirect indicator of nitric oxide (NO) activity, which can influence GFAP protein expression, disrupt the electron transport chain, induce oxidative stress, and lead to cell death.^17,34^ Furthermore, this detrimental cascade is exacerbated by reduced activity of antioxidant enzymes such as SOD, CAT, and GST, ultimately resulting in an excessive accumulation of ROS and the subsequent harmful effects caused by these molecules.

Conversely, inosine exhibits a significant antioxidant effect, effectively reducing levels of ROS and nitrites. This effect may be associated with decreased ATP levels, increased availability of antioxidant compounds such as sulfhydryl groups, and enhanced activity of antioxidant enzymes, including SOD, CAT, and GST. Additionally, it is plausible that inosine’s degradation product, urate, contributes to its antioxidant action by activating the Nrf2 pathway, a key transcription factor that induces endogenous antioxidant defenses and promotes the expression of antioxidant enzymes.^35,36^

### 2.5 Caffeine Modulates the Effect of Inosine on Antioxidant Response but Not on Cytokine Release Following LPS Stimulation

Given that inosine has been reported to exert its effects through the adenosinergic system,^4^ we investigated the potential involvement of adenosine and caffeine, a competitive antagonist of adenosine receptors, in LPS-induced oxidative damage in astrocytes (Figure 7). Caffeine prevented the LPS-induced reduction in SH levels (75%) and in the activities of SOD (24%) and GST enzymes (70%). In contrast, adenosine did not reverse the alterations. However, preincubation with caffeine did not prevent the LPS-induced increase in nitrite levels or the reduction in CAT activity. To elucidate whether the antioxidant effects of inosine are mediated through an adenosine receptor-dependent mechanism, cells were coincubated with inosine and caffeine. Although inosine prevented the increase in nitrite levels (16%), the reduction in SH content (77%), and the reduction in GST activity (64%), caffeine did not modify these effects of inosine. Interestingly, adenosine receptor blockade reversed the antioxidant effect of inosine on SOD (13%) and CAT enzyme activity (55%) (Figure 7).

**Figure 7:**
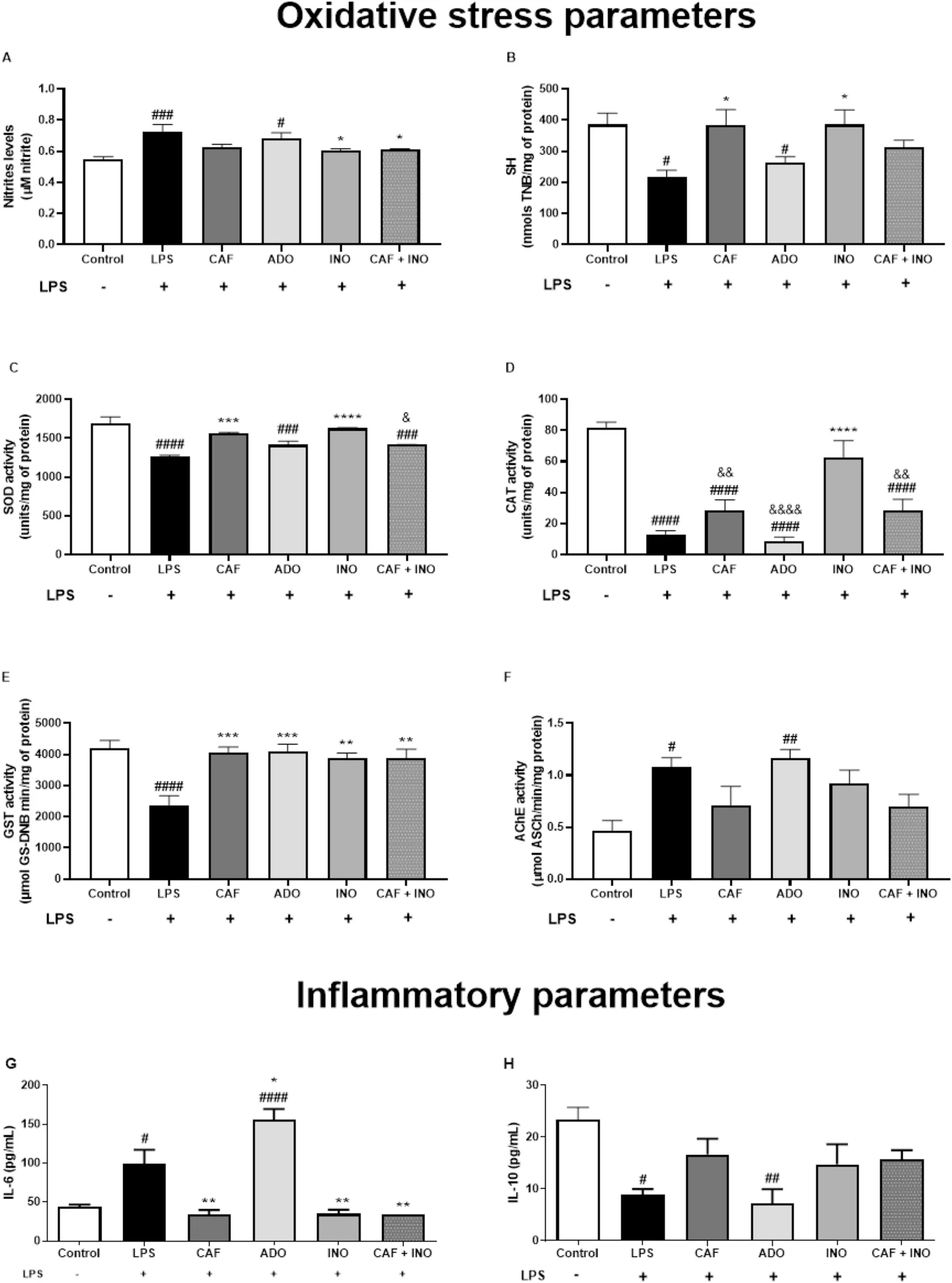
Nitrite levels (A), total sulfhydryl (SH) content (B), superoxide dismutase (SOD) activity (C), catalase (CAT) activity (D), glutathione S-transferase (GST) activity (E), and acetylcholinesterase (AChE) activity (F), interleukin-6 (G), and interleukin-10 (H) in primary astrocyte cultures exposed to LPS (10 μg/mL), inosine (INO, 100 μM), caffeine (CAF, 100 μM), adenosine (ADO, 100 μM), and CAF + INO for 1 h prior to LPS exposure. Cells were subsequently exposed to LPS for 24 h. The experiments were performed in triplicate. Data were analyzed by one-way ANOVA followed by Tukey’s post hoc test and are presented as mean ± SEM ^#, ##, ###, ####^ Significant difference compared to control cells (*P* < 0.05, *P* < 0.01, *P* < 0.001, and *P* < 0.0001, respectively). Cells were subsequently exposed to LPS for 24 h. *, **, ***, **** Significant difference compared to the LPS group (*P* < 0.05, *P* < 0.01, *P* < 0.001, and *P* < 0.0001, respectively). ^&, &&, &&&&^ Significant difference compared to the inosine group (*P* < 0.05, *P* < 0.001, and *P* < 0.0001, respectively).

The exposure of astrocytes to LPS, adenosine, caffeine, and caffeine combined with inosine provided important insights into the involvement of the adenosinergic system in the protective effects of inosine. Although caffeine prevented some of the oxidative and inflammatory alterations induced by LPS, its nonselective antagonistic action and the ability of adenosine to activate multiple receptor subtypes preclude definitive conclusions regarding the participation of specific adenosine receptors.

To further investigate this mechanism, selective A2A receptor antagonists were employed (Table 1). Neither SCH58261 nor istradefylline (IST) alone prevented the LPS-induced reductions in the total thiol content or antioxidant enzyme activities. However, both antagonists abolished the protective effects of inosine on total thiol levels (51%), SOD activity (SCH58261 at 63% and IST at 29%), and CAT activity (SCH58261 at 72% and IST at 85%). These findings provide preliminary evidence that A2A receptor signaling may be associated with the antioxidant-related responses observed under inosine-treated conditions in astrocytes. Importantly, the antagonists alone did not reproduce the protective responses observed with inosine, suggesting that A2A receptor signaling may represent one of the several mechanisms involved in these responses.

**Table 1:** Total Sulfhydryl (SH) Content and SOD and CAT Activities in Primary Astrocyte Cultures Exposed to LPS (10 μg/mL), Inosine (INO, 100 μM), SCH58261 (SCH, 50 nM), Istradefylline (IST, 100 nM), SCH + INO, and IST + INO after 1 h Cells were subsequently exposed to LPS for 24 h^a^

	SH	SOD	CAT
control	357.8 ± 43.7	1731 ± 158.1	76.1 ± 4.9
LPS	106.4 ± 7.4^b^	528.7 ± 62.7^b^	9.0 ± 2.5^b^
INO	298.8 ± 48.1^c^	1599 ± 10.97^c^	67.2 ± 8.0^c^
SCH	172.0 ± 20.5^b^	718.9 ± 96.2^b^	27.7 ± 10.3^b^
SCH+INO	145.8 ± 25.3^b^ ^,^ ^d^	590.3 ± 61.8^b^ ^,^ ^d^	18.6 ± 5.6^b^ ^,^ ^d^
IST	117.0 ± 15.56^b^	584.1 ± 35.3^b^	17.9 ± 7.7^b^
IST+INO	145.7 ± 13.2^b^ ^,^ ^d^	1125.0 ± 187.6^b^ ^,^ ^c^	9.6 ± 1.6^b^ ^,^ ^d^

a Abbreviations: LPS, lipopolysaccharide; INO, inosine; SH, total sulfhydryl content; SOD, superoxide dismutase; and CAT, catalase. Thiol content as nmol TNB/mg protein, SOD and CAT activities as units/mg protein. The experiments were performed in triplicate. Data were analyzed by one-way ANOVA followed by Tukey’s post hoc test and are presented as mean ± SEM.

b There was a significant difference compared to control cells (*P* < 0.05).

c There was a significant difference compared to the LPS group (*P* < 0.05).

d There was a significant difference compared to the INO group (*P* < 0.05).

Additional control experiments demonstrated that caffeine, SCH58261, and IST did not affect astrocyte viability under basal conditions (Figure S1). Likewise, caffeine alone did not alter SH levels or the activities of SOD, CAT, and GST compared to untreated control cells (Table S1).

Finally, to evaluate the LPS-induced inflammatory response in astrocyte cultures, we measured IL-6 and IL-10 levels. Compared to the control cells, LPS stimulation led to an approximately 2.5-fold increase in IL-6 release from astrocytes while significantly reducing IL-10 levels by 2.6-fold. Pretreatment with inosine 1 h before LPS stimulation prevented changes in IL-6 levels but had no effect on IL-10 levels. When cells were coincubated with caffeine, the effects of inosine remained unchanged. Interestingly, adenosine markedly enhanced IL-6 levels by 1.6-fold beyond those observed with LPS exposure alone, while IL-10 levels remained similar to those induced by LPS. Additionally, caffeine, as an adenosine receptor antagonist, prevented the LPS-induced increase in IL-6 levels but did not alter IL-10 levels (Figure 7).

The effects of LPS, adenosine, caffeine, and inosine on cytokine production provide additional insights into the modulation of astrocyte responses under inflammatory conditions. Since caffeine acts as a nonselective adenosine receptor antagonist and adenosine activates multiple receptor subtypes, these findings do not allow definitive conclusions regarding the involvement of specific adenosine receptors in cytokine regulation. Furthermore, the ability of inosine to prevent the increase in IL-6 levels was preserved in the presence of caffeine, suggesting that adenosine receptor activation is unlikely to be the primary mechanism underlying this effect. Taken together, these findings indicate that inosine differentially modulates oxidative parameters and cytokine production in astrocytes, with stronger pharmacological evidence supporting the involvement of A2A receptor signaling in its antioxidant actions than in its effects on IL-6 regulation.

Some limitations of this study are acknowledged. Although the primary cultures were established using a widely employed protocol for obtaining astrocyte-enriched cultures, the culture purity was not directly assessed by immunocytochemical or molecular markers. Therefore, the presence of other glial cells cannot be completely excluded.

In conclusion, inosine protects astrocytes from LPS-induced oxidative damage and disruptions in both cholinergic and purinergic signaling pathways (Figure 8). These findings highlight the glioprotective potential of inosine and demonstrate its ability to attenuate oxidative stress while modulating cytokine production in astrocytes. By preserving redox homeostasis, regulating key enzymes involved in neurotransmission, and preventing the LPS-induced increase in IL-6 levels, inosine emerges as a promising candidate for therapeutic strategies targeting neurodegenerative and neuroinflammatory disorders. Furthermore, the pharmacological findings obtained in this study provide preliminary evidence that adenosinergic signaling, particularly through A2A receptors, may be involved in the antioxidant-related effects observed under inosine-treated conditions, although the underlying molecular mechanisms remain unclear.

**Figure 8:**
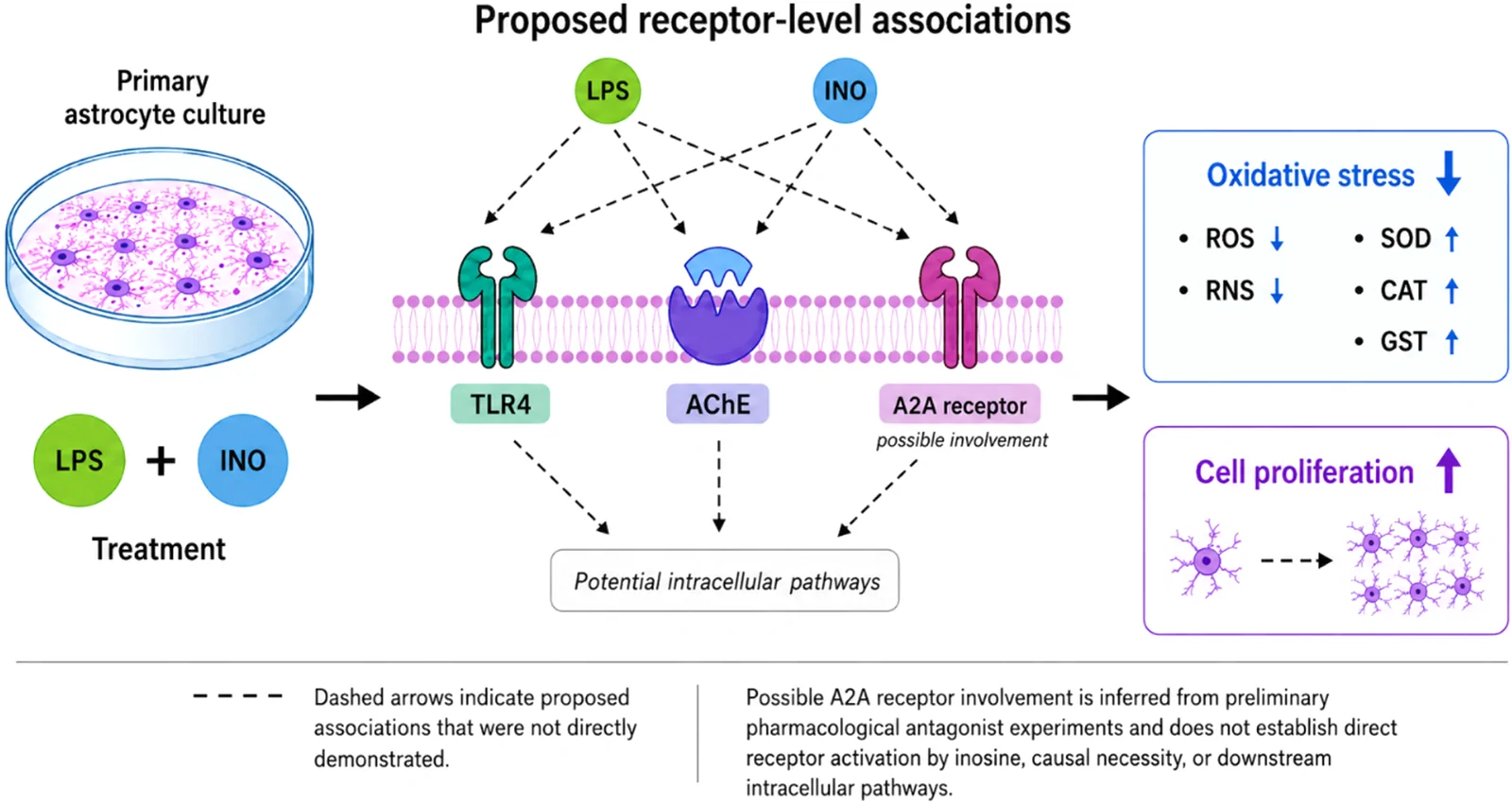
Overview of the main findings observed in LPS-exposed astrocytes under inosine-treated conditions, together with proposed receptor-level associations and potential intracellular pathways.

## 3 Methods

### 3.1 Chemicals

Inosine, lipopolysaccharide (LPS) from*Escherichia coli* (055:B5), 4-(2-hydroxyethyl) piperazine-1-ethanesulfonic acid (HEPES), sodium bicarbonate (NaHCO_3_), 3­(4,5-dimethylthiazole-2-yl)-2,5 diphenyl tetrazolium bromide (MTT), dimethyl sulfoxide (DMSO), sulforhodamine B (SRB), acetylthiocholine iodide (AcSCh), Coomassie Brilliant Blue G, dichloro-dihydro-fluorescein diacetate (DCFH-DA), 5,5′-dithiobis (2-nitrobenzoic acid) (DTNB), caffeine, adenosine (ADO), adenosine monophosphate (AMP), adenosine diphosphate (ADP), istradefylline (IST), SCH58261, adenosine monophosphate (AMP), adenosine diphosphate (ADP), and adenosine triphosphate (ATP) were purchased from Sigma Chemical Co. (St. Luis, MO). Trichloroacetic acid (TCA) and hydrogen peroxide were purchased from Synth (Brazil). Dulbecco’s modified Eagle’s medium (DMEM), fungizone, penicillin/streptomycin, 0.5% trypsin/ethylene diamine tetraacetic acid (EDTA) solution, and fetal bovine serum (FBS) were obtained from Gibco (Gibco BRL, Carlsbad, CA). All other chemicals and solvents used were of analytical grade.

### 3.2 Animals

Neonatal Wistar rats (1–2 days old) were obtained from the Central Animal House of the Federal University of Pelotas (UFPel, Pelotas, RS, Brazil). The animals were housed under controlled environmental conditions (22 ± 2 °C) with a 12 h light/12 h dark photoperiod and had free access to food and water. All experimental procedures involving animals were reviewed and approved by the Ethics Committee of Animal Experimentation of UFPel (protocol no. 4808-2017). Animal handling and experimental procedures were conducted in accordance with the Brazilian Guidelines for the Care and Use of Animals in Scientific Research Activities and with the National Council for the Control of Animal Experimentation.

### 3.3 Primary Astrocyte Culture

Primary astrocyte cultures were obtained from neonatal Wistar rat cerebral cortices according to a previously established protocol.^18^ Cortical tissue was mechanically dissociated in calcium- and magnesium-free balanced salt solution (CMF; pH 7.4), generating a cell suspension that was subsequently centrifuged (at 1000 rpm for 10 min). The resulting pellet was resuspended in DMEM supplemented with 10% FBS (pH 7.6). Cells were plated according to the requirements of each assay. For cytotoxicity analyses, oxidative stress measurements, acetylcholinesterase activity, and purinergic assays, cultures were seeded at densities of 3 × 10^4^, 2 × 10^5^, and 3 × 10^5^ cells per well in poly-l-lysine-coated 96-, 24-, and 6-well plates, respectively. Cultures were maintained at 37 °C in a humidified atmosphere containing 5% CO_2._ Fresh culture medium was supplied every 4 days, and experiments were performed after approximately 20 days. Primary cultures were established using a well-established protocol designed to generate astrocyte-enriched cultures. However, culture purity was not independently verified using immunocytochemical or molecular markers. Therefore, although this protocol is widely used to obtain astrocyte-enriched cultures, the presence of a small proportion of other glial cell types cannot be completely excluded.

### 3.4 Cell Culture Treatment with LPS and Inosine

Inosine was dissolved in water and then diluted in DMEM containing 10% FBS to obtain final concentrations of 12.5, 25, 50, and 100 μM.^20,37,38^ To evaluate cytotoxicity, astrocytes were exposed to inosine for 48 and 72 h. To investigate whether inosine could attenuate LPS-induced astrocyte injury, confluent cultures were first challenged with LPS (10 μg/mL) for 3 h.^20^ Subsequently, the inflammatory medium was supplemented with inosine, and the cultures remained under treatment for an additional 48 or 72 h (Figure 9). This post-treatment design was employed to evaluate the ability of inosine to reverse cellular alterations after the inflammatory insult had been established. The control cells were maintained in DMEM with 10% FBS.

**Figure 9:**
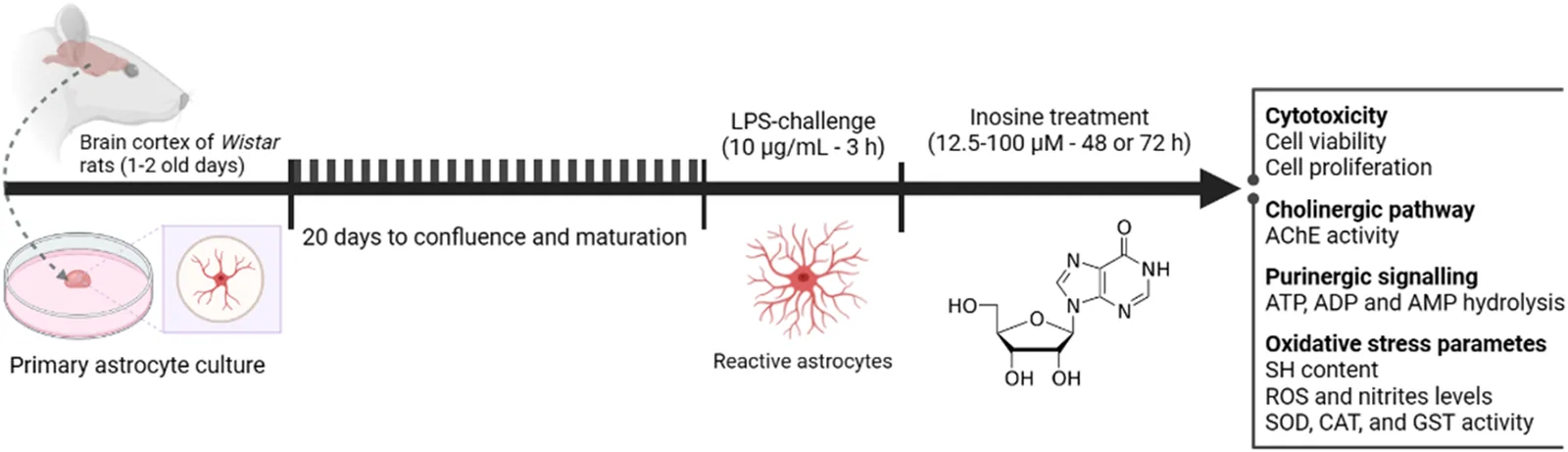
Experimental scheme. Astrocytes were obtained from the cerebral cortex of neonatal Wistar rats (1–2 days old) and maintained under standard conditions for 20 days. Thereafter, cultures were exposed to LPS (10 μg/mL) for 3 h and subsequently treated with inosine for 48 or 72 h.

### 3.5 Investigation of Adenosinergic Pathway

To explore the contribution of adenosinergic signaling, astrocytes were exposed to pharmacological modulators before LPS stimulation. Cultures were incubated for 1 h with caffeine (100 μM; a nonselective adenosine receptor antagonist), SCH58261 (50 nM; a selective A2A receptor antagonist), istradefylline (100 nM; a selective A2A receptor antagonist), adenosine (100 μM), inosine (100 μM), or the respective combinations of inosine with caffeine, SCH58261, or istradefylline. LPS was then added (10 μg/mL) and maintained for 24 h.^39–41^ Caffeine, adenosine, and inosine were dissolved in water, whereas SCH58261 and istradefylline were dissolved in dimethyl sulfoxide (0.05%). After treatment, oxidative stress parameters, acetylcholinesterase activity, and/or cytokine levels were evaluated. This pretreatment design was used to enable receptor modulation before the inflammatory stimulus, allowing the investigation of early adenosinergic signaling events involved in the cellular response to LPS, in contrast to the post-treatment approach applied in the main experimental model to evaluate the therapeutic effects of inosine after the inflammatory insult had been established.

### 3.6 Cytotoxicity

#### 3.6.1 Cell Viability

Cellular metabolic activity, used as an indicator of viability, was determined using the MTT reduction assay.^42^ The cultures were washed with CMF and then incubated with MTT solution (0.5 mg/mL per well) at 37 °C in a humidified 5% CO_2_ atmosphere for 90 min. Subsequently, the medium was removed, and formazan crystals were diluted in DMSO. Finally, the optical density (OD) was measured at 492 nm using a microplate reader (SpectraMax 190). The results were calculated using GraphPad Prism 8.0 software (Prism GraphPad Software, San Diego) and expressed as the percentage of control, following the formula: Cell viability rate (%) = (OD of treated cells/OD of control) × 100%.

#### 3.6.2 Cell Proliferation

Cell proliferation was estimated using the SRB assay, which quantifies total cellular protein content. After the treatment period, culture medium was removed, and the cells were fixed with cold 50% TCA for 45 min at 4 °C. The plates were then washed with distilled water. Fixed cells were stained with 0.4% SRB solution prepared in 1% acetic acid for 30 min at room temperature. Excess dye was removed by repeated washes with 1% acetic acid, and the plates were dried completely. Protein-bound dye was solubilized with 10 mM Tris base (pH 10.5). OD was measured at 530 nm using a microplate reader (SpectraMax 190), and this value was calculated using Prism 8.0 software (Prism GraphPad Software, San Diego) according to the following formula: Cell proliferation rate (%) = (OD of treated cells/OD of control) × 100%.

### 3.7 Preparation of Lysates for AChE Activity and Oxidative Stress Assays

Following the experimental treatments, cultures were rinsed twice with sterile water and harvested by using a cell scraper. Cell suspensions were centrifuged (1000 rpm for 10 min), and the supernatants were collected for biochemical analyses.

### 3.8 Acetylcholinesterase (AChE) Activity

AChE activity was determined using a colorimetric assay based on the hydrolysis of acetylthiocholine, following the method of Ellman et al.^43^ The reaction mixture contained 10 mM DTNB, 100 mM phosphate-buffered saline (PBS; pH 7.5), and 15 μL of cell lysate. After a 2 min preincubation at 27 °C, the reaction was initiated by adding 8 mM acetylthiocholine iodide (AcSCh). The increase in absorbance was monitored at 530 nm for 2 min at 30 s intervals using a SpectraMax 190 microplate reader maintained at 27 °C. Each sample was analyzed in triplicate. Total protein concentration was determined by the Coomassie Brilliant Blue assay using bovine serum albumin as the reference standard,^26^ and enzyme activity was expressed as μmol AcSCh/h/mg of protein.

### 3.9 Oxidative Stress Analysis

#### 3.9.1 Quantification of ROS

Intracellular ROS were quantified using the fluorescent probe 2′,7′-dichlorodihydrofluorescein diacetate (DCFH-DA), as previously described by Dos Santos et al.^44^ Following the experimental treatments, astrocytes were incubated with 1 μM DCFH-DA for 30 min at 37 °C. Fluorescence generated by oxidation of the probe was measured at excitation and emission wavelengths of 485 and 520 nm, respectively. ROS production was normalized to protein concentration and expressed as μmol DCF/mg protein.

#### 3.9.2 Nitrite Levels

Nitrite accumulation in culture supernatants was quantified by the Griess reaction according to Stuehr and Nathan.^45^ Equal volumes of sample and Griess reagent were mixed, and absorbance was measured at 540 nm using a microplate reader (SpectraMax 190). Nitrite concentrations were calculated from a sodium nitrite standard curve and expressed as μM nitrite.

#### 3.9.3 Total Sulfhydryl (SH) Content

The SH content was measured using the DTNB reduction assay according to Aksenov and Markesbery.^46^ In this assay, sulfhydryl groups react with DTNB to generate the yellow compound 5-thio-2-nitrobenzoic acid (TNB). The absorbance of TNB was determined at 412 nm, and SH levels were calculated from the absorbance values and normalized to the protein concentration. Results were expressed as nmol of TNB/mg protein.

#### 3.9.4 Superoxide Dismutase (SOD) Activity

SOD activity was assessed by monitoring the inhibition of adrenaline autoxidation according to the procedure described by Misra and Fridovich.^47^ Changes in absorbance were recorded at 480 nm with a SpectraMax 190 microplate reader, and enzyme activity was normalized to the total protein content. Results were expressed as units/mg of protein.

#### 3.9.5 Catalase (CAT) Activity

Catalase activity was evaluated by measuring the decomposition of hydrogen peroxide according to the method proposed by Aebi.^48^ The decrease in H_2_O_2_ concentration was monitored spectrophotometrically at 240 nm and 37 °C using a SpectraMax 190 microplate reader. Enzyme activity was normalized to protein concentration and expressed as units/mg protein.

#### 3.9.6 Glutathione S-Transferase (GST) Activity

GST activity was determined according to the procedure described by Habig et al.^49^ The assay was based on the enzymatic conjugation of reduced glutathione (GSH) with 1-chloro-2,4-dinitrobenzene (CDNB). The reaction mixture consisted of 20 mM potassium phosphate buffer (pH 6.5), 10 mM GSH, 1 mM CDNB prepared in ethanol, and 20 μL of cell lysate. Enzymatic activity was determined spectrophotometrically and expressed as μmol GS-DNB min/mg of protein.

### 3.10 ATP, ADP, and AMP Hydrolysis

NTPDase activity was determined in a reaction medium containing 112 mM NaCl, 5 mM KCl, 60 mM glucose, 5 mM MgCl_2_, and 50 mM Tris-HCl (pH 8.0), using ATP or ADP (1 mM) as substrates. For 5′-nucleotidase activity, AMP (2 mM) was incubated in a buffer containing 100 mM Tris-HCl (pH 7.5) and 10 mM MgSO_4_. After incubation at 37 °C for 40 min, reactions were stopped with trichloroacetic acid, and the inorganic phosphate released was determined by the malachite green method using KH_2_PO_4_ as the standard. Enzyme activities were normalized to protein concentration and expressed as nmol Pi/min/mg protein.

### 3.11 Determination of IL-6 and IL-10 Levels

Cytokine concentrations (IL-6 and IL-10) were determined in culture supernatants using a commercially available enzyme-linked immunosorbent assay (ELISA) kit (Sigma-Aldrich, St. Louis, MO) according to the manufacturer’s recommendations. Results are expressed as pg/mL.

### 3.12 Statistical Analysis

Statistical analysis was performed using one-way ANOVA followed by Tukey’s post hoc test when appropriate. Data are presented as mean ± SEM. Differences were considered significant at *P* < 0.05 using GraphPad Prism 8.0 software (Prism GraphPad Software, San Diego).

## Supplementary Material



## Data Availability

Please contact the authors for data requests.

## Notes

Figure S1 and Table S1 showing supplementary experimental results; Table S2 containing the raw data corresponding to Table 1. (PDF)

## ∥

Department of Biochemistry and Molecular Biology, Federal University of Santa Maria (UFSM), Santa Maria, RS 97105-900, Brazil
