# Supplementary material for: Inosine Exerts Glioprotective Effects against Lipopolysaccharide-Induced Damage in Primary Astrocyte Cultures: Role of the Adenosinergic System
Source: ACS Omega. 2026 Sep 2;11(36):53798–811. doi: 10.1021/acsomega.6c02414 (PMC13584798; doi:10.1021/acsomega.6c02414)
Supplement: Supplementary file 1 [file ao6c02414_si_001.pdf]

Supplementary Information

Inosine exerts glioprotective effects against lipopolysaccharide-induced damage in primary astrocyte cultures: role of the adenosinergic system

Nathalia S. Pedra[a], Mayara S. S. de Aguiar[a], Fernanda C. Teixeira[a], Julia E. de Mello[a], Natália P. Bona[b], Larissa M. da Silveira[b], Thais M. da Rosa[a], Juliane T. Saraiva[b], William S. Simões[b], Daniela B. R. Leal[c], Rejane G. Tavares[b], Francieli M. Stefanello[b], Roselia M. Spanevello [a,d]*

[a] Center for Chemical, Pharmaceutical and Food Sciences, Graduate Program in Biochemistry and Bioprospecting – Laboratory of Neurochemistry, Inflammation and Cancer, Federal University of Pelotas, Pelotas, RS, Brazil

[b] Center for Chemical, Pharmaceutical and Food Sciences, Graduate Program in Biochemistry and Bioprospecting – Biomarkers Laboratory, Federal University of Pelotas, Pelotas, RS, Brazil

[c] Department of Microbiology and Parasitology - Experimental and Applied Immunobiology Laboratory, Federal University of Santa Maria, Santa Maria, RS, Brazil

[d] Department of Biochemistry and Molecular Biology - Toxicological Enzymology Laboratory, Federal University of Santa Maria, Santa Maria, RS, Brazil

Corresponding author

Roselia Maria Spanevello – Email: roselia.spanevello@ufsm.br

Federal University of Santa Maria. Av. Roraima No. 1000, University City, Camobi District, Santa Maria – RS, 97105-900, Brazil

Contents

Figure S1. Effects of caffeine (CAF, 100 μM), SCH58261 (SCH, 50 nM), and istradefylline (IST, 100 nM) on astrocyte viability. Primary astrocyte cultures were exposed to each compound under the same experimental conditions used in the mechanistic studies. Cell viability was assessed by the MTT assay. No significant differences were observed compared with untreated control cells. Data are presented as mean ± SEM. Statistical analysis was performed using one-way ANOVA followed by Tukey's post hoc test.

Table S1. Effects of caffeine (CAF, 100 μM) on oxidative stress-related parameters in primary astrocyte cultures. Cells were exposed to caffeine under the same experimental conditions used in the adenosinergic pathway experiments. Total sulfhydryl (SH) content and the activities of superoxide dismutase (SOD), catalase (CAT), and glutathione S-transferase (GST) were evaluated. No significant differences were observed compared with untreated control cells. Data are presented as mean ± SEM.

Table S2. Raw data corresponding to Table 1. Total sulfhydryl (SH) content and SOD and CAT activities in primary astrocyte cultures exposed to LPS (10 μg/mL), inosine (INO, 100 μM), SCH58261 (SCH, 50 nM), istradefylline (IST, 100 nM), SCH + INO, and IST + INO after 1 h. Cells were subsequently exposed to LPS for 24 h.

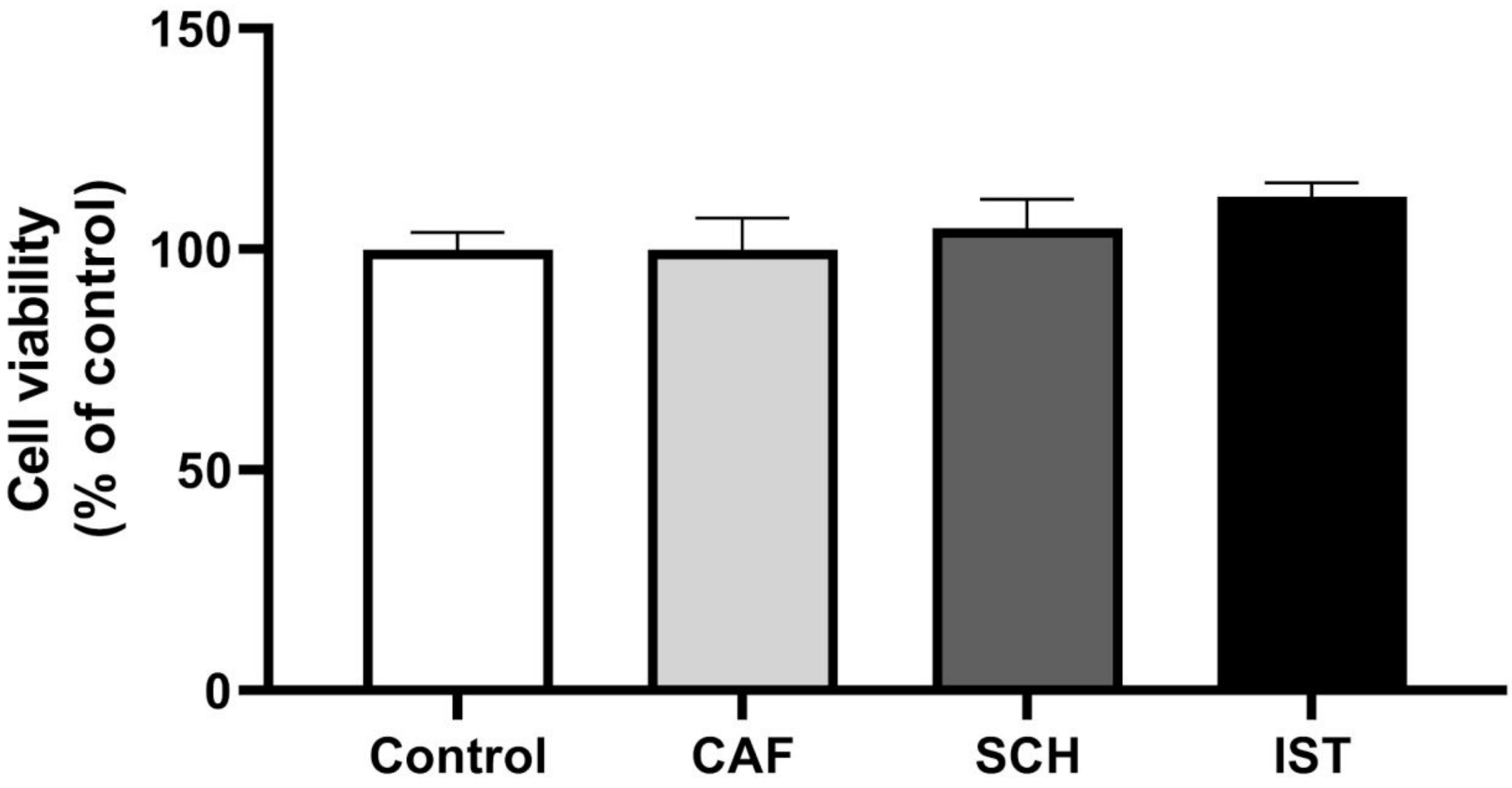

Figure S1. Effects of caffeine (CAF, 100 μM), SCH58261 (SCH, 50 nM), and istradefylline (IST, 100 nM) on astrocyte viability. Primary astrocyte cultures were exposed to each compound under the same experimental conditions used in the mechanistic studies. Cell viability was assessed by the MTT assay. No significant differences were observed compared with untreated control cells. Data are presented as mean ± SEM. Statistical analysis was performed using one-way ANOVA followed by Tukey's post hoc test.

Table S1. Effects of caffeine (CAF, 100 μM) on oxidative stress-related parameters in primary astrocyte cultures. Cells were exposed to caffeine under the same experimental conditions used in the adenosinergic pathway experiments. Total sulfhydryl (SH) content and the activities of superoxide dismutase (SOD), catalase (CAT), and glutathione S-transferase (GST) were evaluated. No significant differences were observed compared with untreated control cells. Data are presented as mean ± SEM.

Group	SH	SOD	CAT	GST
Control	316.6 ± 24.4	1814.0 ± 96.5	75.9 ± 7.0	3339.0 ± 268.6
CAF	337.8 ± 90.3	1946.0 ± 34.91	83.4 ± 8.1	3552.0 ± 180.7

Abbreviations: CAF, caffeine; SH, total sulfhydryl content; SOD, superoxide dismutase; CAT, catalase; GST, glutathione S-transferase. Thiol content as nmol TNB/mg protein, SOD and CAT activities as U/mg protein, and GST activity as μmol GS-DNB min/mg of protein. The experiments were performed in triplicate. Data were analyzed by *t* test and are presented as mean ± SEM.

Table S2. Raw data corresponding to Table 1. Total sulfhydryl (SH) content and SOD and CAT activities in primary astrocyte cultures exposed to LPS (10 µg/mL), inosine (INO, 100 µM), SCH58261 (SCH, 50 nM), istradefylline (IST, 100 nM), SCH + INO, and IST + INO after 1 h. Cells were subsequently exposed to LPS for 24 h.

Experiment	Replicates	Control	LPS	INO	SCH	SCH+INO	IST	IST+INO
SH	1[a]	405.20	93.70	249.71	233.49	223.87	189.43	166.47
		449.47	80.32	215.98	136.20	206.65	120.55	183.61
	2[a]	395.37	103.96	192.08	196.49	97.71	107.87	105.88
		416.18	116.19	243.30	147.37	110.74	77.91	107.75
	3[a]	325.09	110.98	481.57	214.03	164.49	100.84	145.50
		155.48	133.18	410.23	104.41	71.13	105.64	165.16
SOD	1[a]	1475.89	334.08	1517.00	718.88	590.28	584.05	1540.84
		1408.25	336.00	1436.31	894.31	705.60	647.03	1550.82
	2[a]	2207.76	663.73	1372.78	718.88	475.83	643.26	546.75
		2247.92	661.36	1389.81	876.56	688.58	584.05	558.97
	3[a]	1514.53	582.24	1928.01	551.84	590.28	517.41	1262.77
		1533.02	594.91	1952.45	552.81	491.11	528.50	1291.34
CAT	1[a]	67.76	2.365	62.55	59.57	32.95	26.36	11.24
		67.76	5.911	41.70	59.57	39.54	52.72	5.62
	2[a]	79.63	14.04	93.11	18.80	11.46	7.47	9.81
		79.63	2.34	49.00	18.80	9.174	2.49	3.92
	3[a]	97.36	16.98	75.08	5.99	8.507	9.18	13.54
		64.91	12.74	81.91	3.99	10.20	9.18	13.54
